# Global longitudinal strain as an Indicator of cardiac Iron overload in thalassemia patients

**DOI:** 10.1186/s12947-019-0174-y

**Published:** 2019-11-04

**Authors:** Firoozeh Abtahi, Alireza Abdi, Saideh Jamshidi, Mehran Karimi, Mohammad Ali Babaei-Beigi, Armin Attar

**Affiliations:** 1 Department of Cardiovascular Medicine, Shiraz University of Medical Sciences, Shiraz, Iran; 2 Students’ Research Committee, Shiraz University of Medical Sciences, Shiraz, Iran; 3 Hematology Research Center, Shiraz University of Medical Sciences, Shiraz, Iran; 4 Cardiovascular Research Center, Shiraz University of Medical Sciences, Shiraz, Iran

**Keywords:** MRI T_2_^*^, Ferritin, Echocardiography, Speckle tracking, Blood transfusion, Iron overload, Global longitudinal strain

## Abstract

**Background and objective:**

Cardiac involvement due to iron overload is the most common cause of morbidity and mortality in patients with thalassemia, and many patients remain asymptomatic until the late stages. Therefore, early detection of heart problems in such patients at subclinical stages can improve the prognosis of these patients. We investigated the role of speckled tracking (SI) and tissue Doppler echocardiography (TDI) in early detection of iron overload in these patients.

**Methods:**

52 thalassemic patients who were receiving regular blood transfusion with normal global LV function were examined by two- and three-dimensional echocardiography. Cardiac MRI was done and T2* images were considered as the non-invasive gold standard for evaluating cardiac iron deposition. Serum ferritin level was assessed and the relationships between serum ferritin levels and echo finding with cardiac MRI T_2_^*^ was investigated.

**Results:**

No significant relationship was seen between serum ferritin levels and cardiac MRI T_2_^*^. Among the echocardiographic findings, septal systolic myocardial velocity (*P* = 0.002 and *r* = 0.43) and global strain (GLS) (*P* = 0.000 and *r* = 0.60) were significantly associated with T_2_^*^. A GLS < 19.5 could predict a T_2_^*^ level below 20 by 82.14% sensitivity and 86.36% specificity (area under the curve = 0.87; *p* < 0.0001).

**Conclusion:**

While serum ferritin level and ejection fraction are not useful candidates, GLS may be used as a valuable marker to screen thalassemia patients for myocardial iron deposition, using a cut off value below − 19.5. This approach may facilitate the cardiac follow up, reduce the costs, and contribute to preventing deterioration of cardiac function in countries with limited availability of cardiac MRI.

## Introduction

Thalassemia is a microcytic- hypochromic hemolytic anemia due to maternal defects in the synthesis of hemoglobin. About 9% of people around the world carry the gene for β thalassemia. It is the most common genetic disease worldwide [1, 2]. In major thalassemia patients with severe anemia, blood transfusion can relieve clinical manifestations of the disease. Patients with β thalassemia major are transfusion-dependent, and without blood transfusion, they represent severe anemia (hemoglobin less than 6 g/dL) [3]. A common clinical problem in patients with thalassemia major treated with blood transfusions is iron overload. Each blood unit contains 200 to 250 mg of iron [4, 5] which necessitates chelation therapy.

Iron overload acts as a toxin to body organs, particularly the heart, liver and endocrine glands. This problem can be treated with chelation therapy [6]. Cardiomyopathy induced by iron overload was described for the first time in patients with alpha thalassemia in 1964 [7]. In secondary overload, iron metabolism is impaired, and the iron content in the circulation exceeds the transferrin capacity, leading to the emergence of non-transferrin binding iron (NTBI) that is so reactive and leads to the formation of oxygen free radicals. Finally, this leads to the occurrence of membrane lipids peroxidation and oxidative damages to cellular proteins.

Cardiomyopathy induced by iron deposition is a reversible phenomenon provided that iron chelation therapy is started on time. Therefore, early detection of cardiac iron deposition in the prevention of progressive heart failure is vital [8]. Routine examinations such as ECG, clinical examination, typical echocardiography and serum ferritin levels are incapable of the diagnosis of subclinical myocardial involvement. Cardiac MRI T_2_^*^ is a non-invasive and highly sensitive detection method; in fact, it is the gold standard for the diagnosis of cardiac iron overload [9]. However, this method is expensive and not available in many centers.

Conventional echocardiography which examines such factors as Ejection fraction (EF) and FS (Fractional shortening) is not sufficiently accurate for early diagnosis of cardiac iron overload. Previous studies have shown the benefits of newer echocardiographic techniques such as Strain Imaging [10, 11] and Tissue Imaging [12] for the detection of early and subclinical myocardial dysfunction syndromes. However, most of the studies have compared thalassemia patients with normal population and have ignored a comparison with cardiac MRI findings as the noninvasive gold standard [13] and if this comparison is done only a correlation assessment was done. Nevertheless, a more important question is that how these echocardiographic findings differ in patients with a cardiac MRI T_2_^*^ values ​​less than 20 milliseconds as an indicator of myocardial iron deposition. Here, we are going to answer this question and to clarify whether advanced echocardiography can be helpful in thalassemia patients as an available method for diagnosis of subclinical cases cardiac iron overload to identify those in need of further clinical care [14].

## Materials and methods

### Study design and patients

The study was performed from December 22, 2011 to May 19, 2012. The patients with a history of any cardiovascular problems, evidence of dysfunction on echocardiography, valvular diseases, arrhythmias, evidence of CHD, endocrine diseases such as diabetes, hypothyroidism, hypertension, and history of smoking as well as using drugs with an impact on cardiac function were excluded from the study. Accordingly, two patients were excluded from the study due to the evidence of LV dysfunction in echocardiography, and the final sample size reached 50 subjects. A written informed consent was obtained from all patients for this study, and all the study objectives were briefly described for them. In addition, this study was approved by the university medical ethics committee. With an ethical approval number 91–3795.

### Echocardiographic studies

For all patients, echocardiographic studies were performed by GE vivid 9 devices. As patients were lying on the left lateral, subcostal, apical side and para-sternal standard images were taken. All patients were in the sinus rhythm at the time of the study. The factors of Left ventricular end systolic volume (LVESV) and Left ventricular end diastolic volume (LVEDV) were measured, using modified Simpson algorithms and based on two apical 2 chamber and apical 4 chamber views, and the EF was calculated. In tissue Doppler examinations, by placing sample volume in the lateral and mitral septal annulus wall at apical 4 chamber view, the E (early diastolic) systolic s and diastolic velocities were measured on the two walls. Septal strain and lateral strain measurements were performed in the same view. Global longitudinal strain was calculated by 2D specked tracking echocardiography (STE) method at three views of apical 2 chamber, 3 chamber and 4 chamber [10]. Ferritin levels measurement in all cases was done at the hospital’s laboratory at an interval of up to 2 weeks from the time of MRI and echo studies.

### MRI studies

The iron stored in the heart was measured by using T_2_^*^, which is an MRI parameter representing relaxation, and inversely correlated with the iron stored in the heart. MRI examinations were performed, using a 1.5 Tesla Philips scanner. Cardiac T_2_^*^ measurement was done in a “Mid papillary shot axis”. All MRI studies were described by a radiologist; T_2_^*^ values ​​less than 20 ms were reported as abnormal values of myocardial iron. MRI studies were done in the same week that echocardiography was performed.

### Statistical analysis

In this study, Spearman rank correlation test was used to evaluate the correlation between the two methods, and *p* values< 0.05 were considered as significant cases. Receiver-operating characteristic (ROC) curve analysis was done to find a cut off point for GLS based on detection of cardiac iron overload. All statistical analyses were performed, using the Statistical Package for Social Sciences version 17.0 (SPSS Inc., Chicago, IL, USA).

## Results

For this study, 52 asymptomatic patients with thalassemia major (23 females and 29 males) with global LVEF> 55% (Global left ventricular ejection fraction) assessed by echocardiography were selected among thalassemic patients admitted to hospital. They had regularly undergone clinical examination and regular blood transfusion to maintain hemoglobin levels over 9.5 and chelation treatments (Deferoxamine or combination of Deferoxamine and Deferiprone). The patients were 23.7 ± 5 years of age. Serum ferritin level was 2584 ± 19.3 ng/ml and MRI T_2_^*^ was 19.76 ± 10.29 milliseconds.

Spearman’s correlation coefficient was used to investigate the relationship between cardio MRI ${T}_2^{\ast }$ findings with serum ferritin levels and tissue Doppler and Strain echo parameters of these patients. The correlation between MRI ${T}_2^{\ast }$ findings with the serum ferritin levels was not significant (*r* = − 0.14, *p* = 0.92). Other correlations are displayed in detail in Table 1. Also, the patients were divided into two ${T}_2^{\ast }$ groups over and under 20, and their echocardiography findings were compared together (Table 2).

**Table 1:** Echocardiographic findings and their correlation with MRI T_2_^*^ findings and serum ferritin level. Data are represented as mean ± SD

	Echo findings	${T}_2^{\ast }$		Ferritin	
		Correlation coefficient (r)	*P* value	Correlation coefficient (r)	*P* value
EF (%)	58.96 ± 4.96	0.26	0.16	0.12	0.38
Septal systolic Velocity (mm/sec)	5.67 ± 1.03	0.43	0.002	0.12	0.93
Septal early diastolic velocity (mm/sec)	9.63 ± 1.88	0.25	0.08	0.14	0.33
Lateral systolic Velocity (mm/sec)	6.10 ± 1.56	0.25	0.07	0.06	0.65
Lateral early diastolic Velocity (mm/sec)	12.60 ± 1.70	0.25	0.07	0.09	0.51
Septal systolic Strain (%)	1.05 ± 0.26	0.20	0.15	0.14	0.31
Lateral systolic Strain (%)	1.12 ± 0.32	0.11	0.41	0.19	0.18
Global Strain (%)	19.36 ± 3.24	0.60	0.00	0.26	0.06
Left Ventricular End Systolic Volume (mm^3^)	21.85 ± 6.20	0.25	0.07	−0.11	0.42
Left Ventricular End Diastolic Volume (mm^3^)	53.65 ± 12.68	0.21	0.14	−0.02	0.88

**Table 2:** Comparison of Echocardiographic findings between T2* based classified groups. Data are represented as mean ± SD

	${T}_2^{\ast }<20$	${T}_2^{\ast }>20$	*P* value
EF (%)	59.32 ± 4.9	58.5 ± 5	0.59
Septal systolic Velocity (mm/sec)	5.27 ± 0.89	6.18 ± 0.10	0.20
Septal early diastolic velocity (mm/sec)	9.26 ± 1.13	10.10 ± 1.99	0.61
Lateral systolic Velocity (mm/sec)	5.98 ± 1.39	6.38 ± 1.78	0.12
Lateral early diastolic Velocity (mm/sec)	12.5 ± 1.70	12.68 ± 1.74	0.77
Septal systolic Strain (%)	1.01 ± 0.25	1.10 ± 0.26	0.20
Lateral systolic Strain (%)	1.08 ± 0.31	1.16 ± 0.34	0.40
Global Strain (%)	17.64 ± 2.56	21.55 ± 2.68	< 0.001
LVESV.BS (mm^3^)	20.58 ± 5.38	23.46 ± 6.90	0.10
LVEDV.BS (mm^3^)	51.25 ± 11.58	56.70 ± 31.13	0.13
Ferritin Level (ng/ml)	2591.50 ± 1920.45	2575.27 ± 1927.46	0.98

Among echocardiographic parameters, septal myocardial velocity and global strain had a significant relationship with ${T}_2^{\ast }$ (*P* = 0.002, *P* < 0.001); no significant association was found among the other factors.

**Fig. 1:**
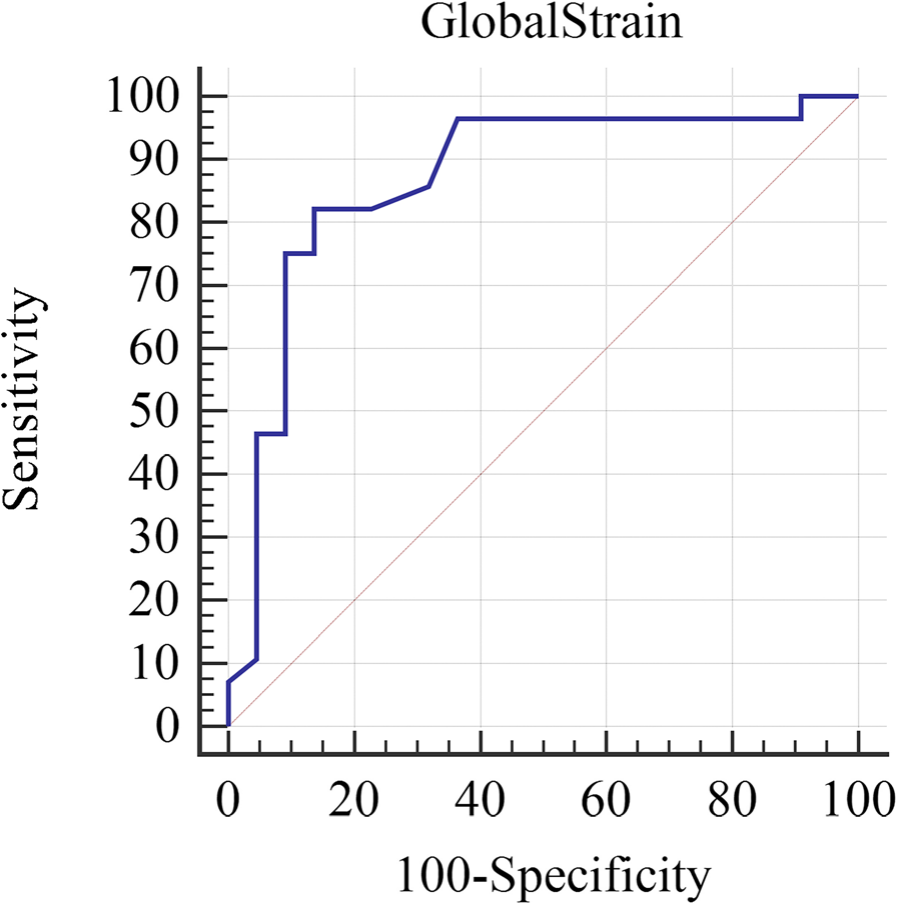
Receiver-operating characteristic (ROC) curves for global longitudinal strain (GLS) and magnetic resonance imaging (MRI) T2* value above 20 milliseconds from below 20 milliseconds as an indicator of cardiac iron deposition. As is shown taking a GLS below −19.5% as the threshold could predict a T_2_^*^ level below 20 milliseconds with a sensitivity of 82.14% and specificity of 86.36% (area under the curve [AUC] = 0.87, *p* < 0.0001

A global strain rate < 19.5 could predict a T_2_^*^ level below 20 with a sensitivity of 82.14% and specificity of 86.36% (area under the curve [AUC] = 0.87, *p* < 0.0001; Fig. 1).

## Discussion

Here, we evaluated the correlation of serum ferritin level or echo Doppler and strain echo parameters of thalassemia patients with the findings of MRI T_2_^*^ as the gold standard for cardiac iron deposition. Among the variables measured, serum ferritin level and ejection fraction were not significantly associated with MRI T_2_^*^ findings, which indicates their inefficiency in predicting abnormal deposition of cardiac iron as well as early diagnosis of heart failure in patients with thalassemia. Among the echocardiographic findings, septal systolic myocardial velocity and global longitudinal strain had a highly significant relationship with T2*, but there was no significant association in the rest of them. A global strain rate < 19.5 could predict a T2* level below 20 with a sensitivity of 82.14% and specificity of 86.36%.

Several studies have evaluated the correlation of various echocardiographic parameters with those from cardiac MRI in thalassemia patients. In a study conducted by Aypar et al. on 33 patients with thalassemia, sepal SM (Septal systolic myocardial velocity) and septal EM (Septal early diastolic myocardial velocity) showed a significant relationship with T_2_^*^ [15]. In another study on 30 patients with thalassemia conducted by Magri et al. [16], among the SI findings, systolic strain of RV free wall, systolic strain of the septal wall, and systolic strain of lateral wall were significantly associated with T_2_^*^. Also, from TDILVSM (Left ventricular lateral wall systolic myocardial velocity) findings, the factors of RVEM (Right ventricular early diastolic myocardial velocity), septal EM, septal SM and LVEM (Left ventricular lateral wall early diastolic myocardial velocity) showed a significant correlation with T_2_^*^. These findings are in agreement with those of our study which indicates that septal systolic myocardial velocity is correlated with the T2* value. The probable reason for variations in diastolic myocardial function in the septal wall and RV free wall compared to the Lat LV wall is increased iron deposition at the septal wall and thinner RV free wall [16]. As iron deposition is very common in the septal part of LV, this part could be expected to have lower strains under conditions of cardiac iron overload.^13^

Comparison of echocardiographic parameters between normal population and thalassemia patients was the subject of some studies. In a study carried out by Hamdy on 27 thalassemia patients and 14 control subjects, the results of SI and TDI showed that thalassemic patients had regional systolic dysfunction in the LV lateral wall, diastolic dysfunctions in the LV septal wall, and RV free wall compared with the control group [17]. In a study conducted by Vogel et al., 52 asymptomatic thalassemia patients were examined with TDI and MRI T_2_^*^, where thalassemic patients had lower systolic and diastolic myocardial velocities compared to the control group [18]. In this study, 73% of the patients had abnormal cardiac iron overload and 87% had regional systolic and diastolic dysfunctions. The TDI sensitivity and specificity for the detection of abnormal iron deposits were 88 and 65%, respectively [19]. In another study by Bay et al., it was shown that in children with thalassemia there is a larger left ventricular end-systolic diameter, end-diastolic and end-systolic volume, left ventricular mass index, and mitral early/late diastolic flow velocity ratio (*p* < 0.05). Strain and strain rate imaging study of the basal lateral wall of the left ventricle was higher in patients than in controls. They concluded that LV volume and mass index parameters might be more sensitive than the other conventional and strain/strain rate imaging parameters during childhood. However, the adulthood strain and strain rate imaging values may be lower than those of the controls [20, 21]. Parsaee and colleagues have shown than STI is helpful for detecting early stages of left ventricular dysfunction in thalasemic patients [14]. In their study, they noticed that there was a significant reduction in GLS (− 20.9% ± 1.9 vs. -22.2 ± 1.03) and also basal segments longitudinal strain compared to normal subjects group (− 17.4% ± 2.7 vs. -19.6% ± 1.2). They noticed that circumferential strain is not associated with left ventricular dysfunction. However, Ari et al. have noticed that an abnormal strain value, especially circumferential, may be detected as the first finding of abnormal iron load and related to T2* values [22].

However, despite the importance of the mentioned findings, the most important correlations to investigate, are those with cardiac MRI T_2_^*^ values ​​less than 20 milliseconds as an indicator of myocardial iron overload. As is shown in Table 2, among all the parameters evaluated in our study (including ferritin level), only GLS showed a significant correlation with a cardiac MRI T_2_^*^ values ​​less than 20 milliseconds. In agreement with our findings, in a study conducted by Silviarat et al. on 31 thalassemic patients with normal global LVEF, it was shown that serum ferritin levels could not reveal the iron deposition in the myocardium [19]. In another study by Garceau et al. on 45 patients with thalassemia major or Diamond Blakfan anemia who were receiving chronic blood transfusion, a strong and direct logarithmic relationship was found between global longitudinal strain and T_2_^*^, and it was concluded that the global longitudinal strain can identify T_2_^*^ under 20 with a sensitivity of 76% and specificity of 88% [23]. Also, Pizzino and colleagues have shown that GLS showed a significant correlation with T2* values (*R* = − 0.49; *P* = 0.001) and it was significantly lower in patients with a T2* value lower than 20 milliseconds (− 18.3 ± 2 vs. − 21.3 ± 2.7, *P* = 0.02). In fact, patients with impaired GLS (<− 19.5%) had a significant higher risk of showing significant cardiac iron deposition (Odds-ratio-OR = 17; 95%) [1]. In our study, GLS had a statistically significant correlation with T2* values and when taking a threshold of 19.5 as the cut off value, it could detect Iron deposition with a sensitivity of 82.14% and specificity of 86.36%. Accordingly, it may be suggested that assessment of GLS can be used as a useful and less expensive tool for screening myocardial iron overload, especially in countries with a limited MRI availability for logistic and economic reasons.

### Limitations

This study had some limitations. Here, we did not include a control normal group in our study and, consequently, we could not find any specific cut off points for GLS to discriminate cardiac iron overload condition from its absence. In addition, our study was a cross- sectional one and we could not evaluate the sensitivity of GLS for early detection of cardiac iron overload during long term monitoring and follow ups.

## Conclusion

According to the studies’ results mentioned and considering the significant relationship found in our study between global longitudinal strain and septal systolic myocardial velocity with T_2_^*^, one can conclude that GLS may be a suitable means to detect Iron deposition in thalassemia major patients. Future prospective studies are needed to find out whether this modality can serve as a screening tool for early detection of myocardial iron overload.

## Data Availability

The datasets generated and/or analyzed during the current study are available from the corresponding author on reasonable request.

## Abbreviations

AUC: Area under the curve
CHD: Coronary heart disease
ECG: Electrocardiography
EF: Ejection fraction
FS: Fractional shortening
GLS: Global strain
LV: Left ventricle
LVEDV: Left ventricular end diastolic volume
LVEF: Left ventricular ejection fraction
LVEM: Left ventricular lateral wall early diastolic myocardial velocity
LVESV: Left ventricular end systolic volume
NTBI: Non-transferrin binding iron
ROC: Receiver operating characteristic
RV: Right ventricle
RVEM: Right ventricular early diastolic myocardial velocity
sepal SM: Septal systolic myocardial velocity
septal EM: Septal early diastolic myocardial velocity
SI: Speckled tracking
STE: Speckle tracking echocardiography
TDI: Tissue Doppler echocardiography
TDI: Tissue Doppler imaging
TDILVSM: Left ventricular lateral wall systolic myocardial velocity
